# Assessment of Lung Disease in Finishing Pigs at Slaughter: Pulmonary Lesions and Implications on Productivity Parameters

**DOI:** 10.3390/ani11123604

**Published:** 2021-12-20

**Authors:** Yania Paz-Sánchez, Pedro Herráez, Óscar Quesada-Canales, Carlos G. Poveda, Josué Díaz-Delgado, María del Pino Quintana-Montesdeoca, Elena Plamenova Stefanova, Marisa Andrada

**Affiliations:** 1 Veterinary Histology and Pathology, Institute of Animal Health and Food Safety (IUSA), Veterinary School, University of Las Palmas de Gran Canaria, 35416 Las Palmas de Gran Canaria, Spain; o.quesada@ulpgc.es (Ó.Q.-C.); josue.diazdelgado@tvmdl.tamu.edu (J.D.-D.); elena.plamenova101@alu.ulpgc.es (E.P.S.); marisaana.andrada@ulpgc.es (M.A.); 2 Departament of Comparative Pathology, Veterinary School, University of Las Palmas de Gran Canaria, 35416 Las Palmas de Gran Canaria, Spain; 3 Epidemiology and Preventive Medicine, Institute of Animal Health and Food Safety (IUSA), Veterinary School, University of Las Palmas de Gran Canaria, 35416 Las Palmas de Gran Canaria, Spain; c.g.povedaturrado@reading.ac.uk; 4 Texas A&M Veterinary Medical Diagnostic Laboratory, Pathology Division, College Station, TX 77843, USA; 5 Departament of Mathematics, University of Las Palmas de Gran Canaria, 35017 Las Palmas de Gran Canaria, Spain; mariadelpino.quintana@ulpgc.es

**Keywords:** lung lesions, *Mycoplasma hyopneumoniae*, porcine circovirus type 2, porcine reproductive and respiratory syndrome virus, serology, swine pathology

## Abstract

**Simple Summary:**

Examination of lung lesions at slaughterhouses provides important information regarding swine respiratory disease presence in farms worldwide. This study evaluated pulmonary lesions in pigs at slaughter and assessed their effect on productive parameters. We observed a high occurrence (73.1%) of lung lesions in a cohort of 108 pigs; these lesions were associated with primary bacterial disease or a combination of bacterial and viral pathogens. The animals with more severe lesions had lower weight gain, remained at the farm longer, and were exposed to reinfection. Through laboratory tests, we demonstrated coinfections between *Mycoplasma hyopneumoniae*, porcine circovirus type 2, and porcine reproductive and respiratory syndrome virus in affected lungs. We suggest that pigs that do not reach the desired weight at slaughter age should be sent to slaughter regardless, thus avoiding economic losses due to suboptimal productive parameters and aggravated respiratory disease by reinfection.

**Abstract:**

Swine respiratory disease is associated with productive losses. We evaluated the prevalence of lung lesions with an emphasis on *Mycoplasma hyopneumoniae* (Mh), porcine circovirus type 2 (PCV2) and porcine reproductive and respiratory syndrome virus (PRRSV), as well as the impact on productive parameters in 108 finishing pigs at slaughter. Pathologic, immunohistochemical (IHC) and serologic analyses were performed. Pneumonic processes were observed in 73.1% of the animals. They mainly consisted of cranioventral bronchopneumonia (CBP) (46.3%) and pleuritis (17.6%). Microscopically, bronchointerstitial pneumonia (67.4%) was common and was occasionally combined (27.9%) with interstitial pneumonia (IP). Mh and PCV2-antigens were detected in bronchointerstitial pneumonia (70.7%) and IP cases (33.3%). There were low titers against Mh (18%) and high titers against PRRSV (100%) and PCV2 (65%). Animals with CBP remained at the farm longer; those with >10% of lung parenchyma involvement were sent later (208.8 days old) and had a lower average carcass weight (74.1 kg) and a lower daily weight gain (500.8 gr/day) compared with animals without lesions (567.2 gr/day, 77.7 kg, 200.8 days old). We suggest that animals that do not reach the weight at slaughter should be sent to slaughter regardless to avoid further negative impacts of respiratory disease in productive parameters.

## 1. Introduction

The European Union (EU) is the world’s second largest producer of pig. From May–June 2021, EU livestock was estimated at 130 million heads, representing a decrease of 0.5% with respect to May–June 2020. Spain is the second leading producer in the EU, with almost 32.5 million heads, along with Germany and France, who both comprise half of the EU’s total production [1]. In 2020, around 56 million heads were slaughtered in Spain, with a total carcass weight of 5 million tons. In the Canary Islands, the swine sector is minor compared with that in other Spanish Autonomous Communities, with over 65,012 heads slaughtered in 2020 [2].

Swine respiratory diseases have had considerable economic impact over intensive pig farming worldwide, causing high morbidity and mortality, increased medication cost, reduced feed conversion and growth rate, and lower carcass quality [3,4,5,6]. Respiratory disease in swine is multifactorial and is often the result of the interplay of infectious agents and environmental factors, among others [7,8,9,10]. Production system type, all in–all out management, ventilation, and flooring type are some of the noninfectious causes that significantly contribute to the development of respiratory disease [8]. These risk factors can create, either by increasing transmission or spread of pathogens, stressful conditions for the animal or direct damage to the respiratory tract [7,8,11]. Pneumonia is common in pigs at the slaughterhouse, and the prevalence may range between 19% to 79%, depending on country, region, production system, or lung lesion scoring system used [8,11,12,13,14]. Pneumonic lesions can be classified according to gross morphologic patterns. Prevalent pneumonic patterns in swine worldwide are bronchopneumonia, interstitial pneumonia and embolic pneumonia. Bronchopneumonia is characterized by cranioventral consolidation of the lungs and can be subclassified into suppurative or fibrinous bronchopneumonia. Interstitial pneumonia is often widely distributed throughout the lungs, and affected lung lobes may fail to collapse and may have a rubbery consistency. Embolic pneumonia is characterized by the presence of firm, discolored, randomly scattered foci in multiple lobes caused by hematogenously disseminated bacterial emboli [15].

Diagnosis of respiratory disease in pigs is often challenging and should rely on the combination of gross evaluation of lung lesions at the slaughter line, histopathologic analysis, and ancillary laboratory tests, e.g., culture, polymerase chain reaction (PCR), immunohistochemistry (IHC), enzyme-linked immunosorbent assay (ELISA), immunofluorescence (IF), in situ hybridization (ISH). Some of the common scoring systems applied to evaluate and monitor swine respiratory disease at slaughter are the methods proposed by Madec and Kobish (1982) [16], Christensen et al. (1999) [17], and Piffer and Brito (1991) [18]. Although these may have methodologic differences and respective strengths and weaknesses, these are important tools that allow for estimating the severity of clinical and subclinical infections [3,6].

*Mycoplasma hyopneumoniae* (Mh) is a common and widespread bacterial respiratory pathogen associated with lung lesions in pigs [8]. Mh often plays a major role in porcine respiratory disease complex (PRDC), together with porcine reproductive and respiratory syndrome virus (PRRSV), porcine circovirus type 2 (PCV2), swine influenza virus (SIV), *Pasteurella multocida*, *Actinobacillus pleuropneumoniae* (App), *Streptococcus suis* and *Glaesserella parasuis* [11,14,19,20,21]. The aim of this study was to evaluate the prevalence of lung lesions with an emphasis on Mh, PCV2, and PRRSV interplay and their impact on productive parameters, namely carcass weight and average daily weight gain (DWG) in a cohort of naturally infected farming pigs at slaughter.

## 2. Materials and Methods

### 2.1. Study Design

A total of 108 hybrid-breed pigs from a commercial farrow-to-finish herd with 250 sows located in Teror (altitude 548.66 m), Gran Canaria (Spain), were evaluated. The farm had a northeast orientation, with a local annual average temperature of 19.8 °C. This farm used an all in–all out management by pen. Farrowing and weaning pens had forced ventilation and temperature control, while the fattening phase had natural ventilation without temperature control. The animals were randomly chosen at weaning, including neutered males and females of different litters born the same day. The piglets were identified by ear marking. The herd’s vaccination protocol included a commercial Mh monodose vaccine in 3-week-old piglets and an *Erysipelothrix rhusiopathiae* and porcine parvovirus commercial vaccine (as per manufacturer’s recommendations).

### 2.2. Pathologic and Immunohistochemical Examinations

#### 2.2.1. Gross Examinations at Slaughterhouse

Animals (*n* = 108) were slaughtered at the “insular slaughterhouse” in Gran Canaria. All animals submitted for slaughter had reached finishing weight. The lungs were retrieved from the carcasses and examined ex situ. The percentage (%) of lungs with pneumonic lesions was determined, and the morphologic patterns of pneumonia [6] were recorded. These included (a) cranioventral bronchopneumonia (CBP), (b) fibrinonecrotic pneumonia (FNP), (c) interstitial pneumonia (IP), (d) embolic pneumonia (EP), (e) granulomatous pneumonia (GP), and (f) pleuritis. CBP is characterized by purple to gray areas of pulmonary consolidation, often located in the apical, intermediate, accessory and cranial parts of the diaphragmatic lobes. FNP is characterized by dark and solid foci with or without fibrinous pleuritis, often involving focal and demarcated lesions, mainly in the cardiac and apical lobes. IP is characterized by failure to collapse, firmness, mottling, and consolidation. EP is characterized by multifocal, small, white foci with a red halo, often distributed randomly throughout the lung parenchyma. GP is characterized by circumscribed, variably sized, firm, caseous or noncaseous nodules, randomly distributed throughout the lungs. Pleuritis is characterized by the presence of fibrinous or fibrous adhesions between the visceral and parietal laminae of the pleura. To assess the severity of the lesions, the percentage of affected pulmonary parenchyma was quantified, applying correction factors for each lobe, according to the method by Piffer and Brito (1991) [18]. Five categories were defined: group 1, lungs with lesions affecting between 0.1% and 10% of the parenchyma; group 2, from 10.1% to 25%; group 3, 25.1% to 50%; group 4, 50.1% to 75%; and group 5, 75.1% to 100%.

#### 2.2.2. Histopathologic Examination

Lung lobes from 50 animals with some pneumonic pattern were randomly sampled for histopathologic and immunohistochemical examinations. Samples were taken from each affected lobe, including transition areas between diseased and healthy regions. The location of the lesions varied among the animals, sampling mainly the cranial right lobe. These samples were fixed in 10% neutral buffered formalin, embedded in paraffin wax, and 5 µm sections were stained with hematoxylin and eosin. Lung tissue sections were examined systematically in detail, with special emphasis on bronchi, bronchioles, bronchi/bronchiole-associated lymphoid tissue (BALT), alveoli, peribronchial, peribronchiolar and interlobular connective tissues, and pleura. Bronchointerstitial pneumonia lesions were graded by following the method proposed by Livingston et al. (1972) [22]. Briefly, (-) no lesions; (I) one or more lymphoid nodules (BALT) involving the muscularis mucosae of bronchi and bronchioles; (II) lymphoid nodules affecting the muscularis mucosae of bronchi and bronchioles and presence of inflammatory cells in the septal wall, bronchus, and alveolar lumen; (III) perivascular and peribronchiolar hyperplasia of BALT with inflammatory cells in the alveolar septa and neutrophils in the bronchial and alveolar lumens; and (IV) marked and extensive perivascular and peribronchiolar BALT hyperplasia.

#### 2.2.3. Immunohistochemical Analysis

For IHC, primary antibodies included: a polyclonal anti-Mh antibody (Strain J; 1:600 dilution; provided by Dr N.C. Feld, Danish Veterinary Diagnostic Laboratory, Copenhagen V, Denmark), a monoclonal anti-PRRSV antibody (1AC7; 1:250 dilution; Ingenasa, Madrid, Spain), and a monoclonal anti-PCV2 antibody (36A9; 1:200 dilution; Ingenasa, Madrid, Spain). Endogenous peroxidase activity was blocked by incubation with 0.3% hydrogen peroxide in methanol for 30 min at room temperature. Sections were then treated with pronase (Sigma-Aldrich Corporation, St. Louis, MO, USA.) 0.1% in phosphate-buffered saline for 5 min at room temperature (Mh) or 8 min at 37 °C (PRRSV). Primary antibodies were applied overnight at 4 °C. Biotinylated rabbit anti-mouse serum (DAKO, Glostrup, Denmark) for monoclonal antibodies or swine anti-rabbit serum (DAKO, Glostrup, Denmark) for polyclonal antibodies, diluted 1:20 for PRRSV and PCV2, and 1:200 for Mh, were used as secondary reagents. The avidin–biotin–peroxidase complex (Vector Laboratories Inc., Burlingame, CA, USA) method was used in a dilution of 1:50. Immunoreactivity was visualized by red chromogen 3-amino-9-ethylcarbazole (Sigma-Aldrich Corporation, St. Louis, MO, USA), and sections were counterstained with Mayer’s hematoxylin (DAKO, Glostrup, Denmark).

### 2.3. Serologic Analysis

#### 2.3.1. Blood Collection and Sera Preparation

A longitudinal serum profile from 22 animals was developed by bleeding at 38, 77 and 136 days, using vacutainer^®^ tubes (5 mL) with no additive. Twenty percent of the animals included in the study (*n* = 108) were randomly chosen, including piglets of each litter. These animals were vaccinated with a Mh monodose vaccine at 21 days old, such that the blood collection coincided with 17, 56 and 115 days post-vaccination (dpv). Sera samples were harvested by centrifugation for 3 min at 4136 relative centrifugal force and stored at −20 °C until testing.

#### 2.3.2. ELISA Tests

Presence of antibodies against Mh, PCV2 and PRRSV were analyzed using commercial ELISA tests for porcine sera samples. Blocking ELISA (INGEZIM M.HYO COMPAC test kit, Ingenasa, Madrid, Spain) was used to detect anti-Mh antibodies. Double recognition ELISA (INGEZIM PRRS DR test kit, Ingenasa, Madrid, Spain) was used to detect antibodies against PRRSV. Specific anti-PCV2 immunoglobulin (Ig) G and IgM antibodies were detected using capture ELISA (INGEZIM Circovirus IgG/IgM test kit, Ingenasa, Madrid, Spain). The latter allowed for elucidating chronology of infection (i.e., active, recent or old infection). Optical densities (ODs) were measured by an Anthos 2020 version 1.8up ELISA reader (450 nm filter). Controls and samples were processed by duplicate, and the average OD value was considered. Validation and interpretation of the results was performed according to manufacturers’ recommendations.

### 2.4. Evaluation of Productive Parameters

Each animal was weighed at 21 days old and at the beginning of the finishing phase (77 days old). The end of the finishing phase was established based on farmer’s criteria, considering an optimal body weight at slaughter for each animal. The minimal time at this phase was 14 weeks, and no animal was held for longer than 21 weeks. Animals had free access to water and were fed ad libitum. Feeding intake control per week was registered, as well as the weight gain for each pen during the whole study. Finally, age and carcass weight of each animal were measured at slaughter time. The average daily weight gain (DWG) for each animal was calculated following the equation employed by Ferraz et al. (2020) [5].

### 2.5. Statistical Analyses

The prevalence of animals with lung lesions (animals with lung lesions/total animals) was calculated. Productive parameters such as weight gain (weight at slaughter—weight at weaning) and average daily weight gain (DWG) ((weight at slaughter—weight at weaning)/days on finishing phase) were also measured.

Statistical analysis of data was performed by IBM SPSS Statistics 27 (IBM Corp. Released 2020. IBM SPSS Statistics for Windows, Version 27.0. Armonk, NY, USA: IBM Corp). Categorical variables were summarized using percentages and relative frequencies. The equality of proportions of the categories contrasted with the non-parametric binomial and Chi-square tests. Mean and standard deviation summarized the numerical variables. To analyze the samples’ normality, the Kolmogorov–Smirnov and Shapiro–Wild tests were used. Student’s t-test or the non-parametric Mann–Whitney U test were used to compare two means of the independent samples. Three means of independent samples were compared using the one-way ANOVA procedure or the non-parametric Kruskal–Wallis test. The results were considered statistically significant if *p* value < 0.05.

## 3. Results

### 3.1. Pathologic and Immunohistochemical Examinations

#### 3.1.1. Gross Examinations at Slaughterhouse

**Figure 1:**
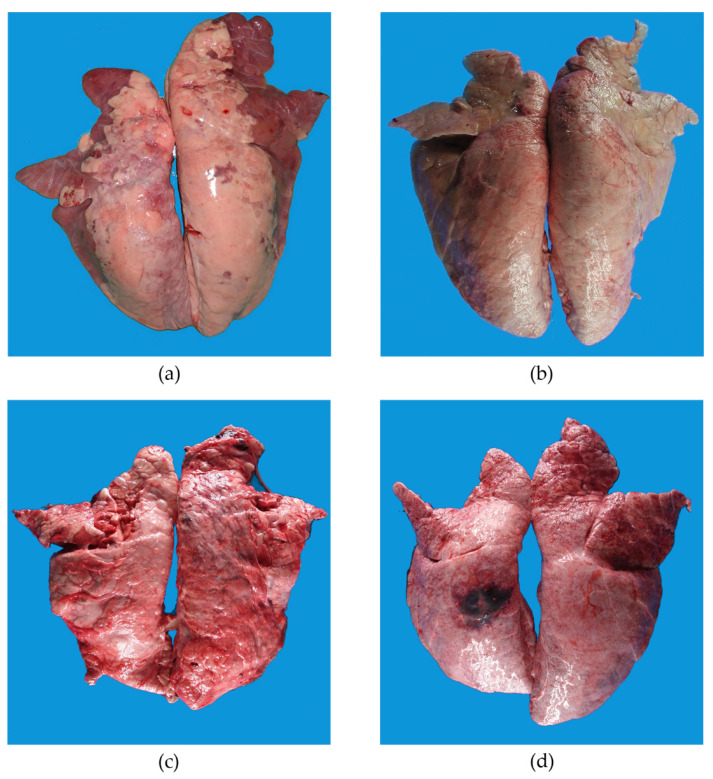
Gross lung lesion patterns observed in pigs at slaughter. (**a**) Acute CBP lesions are characterized by cranio-ventral pulmonary consolidation. (**b**) Chronic CBP lesions are characterized by pale pink to gray cranioventral consolidation. (**c**) Pleuritis. Extensive pleuritis throughout the dorsal aspect of the lung lobes. (**d**) Focal pleuritis associated with fibrinonecrotic and hemorrhagic pneumonic focus on the dorsal aspect of the left caudal lung lobe.

Grossly, 73.1% (79/108) of the animals had some pneumonic pattern. Pneumonic lesions included 46.3% (50/108) CBP, 1.9% (2/108) IP, 6.5% (7/108) FNP, 0.9% (1/108) EP, and 17.6% (19/108) pleuritis (Figure 1). The average percentage of pulmonary parenchyma affected was 12.8%. Animals with lung lesions (*n* = 79) had an average of 17.5% pulmonary parenchyma affected. Nearly half of the animals (48.1%; 38/79) with lung lesions had <10% of affected pulmonary parenchyma, 29.1% (23/79) of the animals had 10.1% to 25% of parenchymal involvement, seven (8.9%) animals had 25.1% to 50%, two (2.5%) animals had 50.1% to 75%, and six (7.6%) animals had 75.1% to 100%. Lung score difference between this group was statistically significant (*p* < 0.001). Statistical analyses results are summarized in Table 1. In terms of pneumonic patterns, animals with CBP had 16.4% of affected lung parenchyma, with the right lung lobe more affected than the left one (9.6 vs. 6.8%). The percentage of affected lung parenchyma was 25.3% in pleuritis cases, 8.5% in FNP cases, 3.3% in IP, and 15.3% in EP cases.

#### 3.1.2. Histopathologic Examination

**Figure 2:**
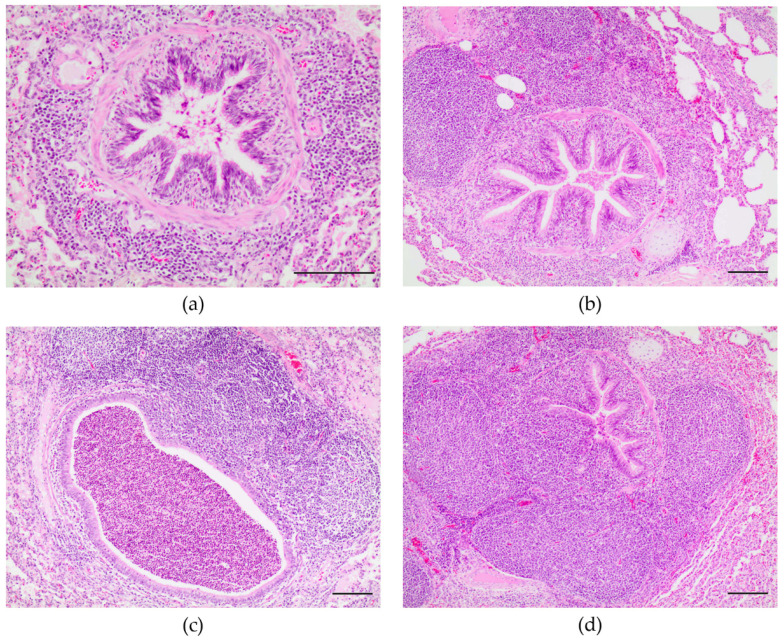
Microscopic features of bronchointerstitial pneumonia compatible with Mh infection in pigs at slaughter. Lesions are characterized by peribronchial and perivascular lymphocytic hyperplasia with pleocellular inflammatory cells in the alveolar septa and neutrophils within the bronchial and alveolar lumens. (**a**) Grade I (magnification 200×). Scale, 100 µm. (**b**) Grade II (magnification 100×). Scale, 100 µm. (**c**) Grade III (magnification 100×). Scale, 100 µm. (**d**) Grade IV (magnification 100×). Scale, 100 µm. Hematoxylin and eosin stain.

Lung samples were taken from 50 animals (43 with gross CBP lesions, 4 with FNP and 1 with IP, EP and pleuritis, each). A total of 95.3% (41/43) of the animals sampled with gross CBP lesions exhibited histologic findings consistent with Mh-bronchointerstitial pneumonia. These were characterized by pleocellular inflammatory infiltrates, including macrophages and neutrophils within the bronchi, bronchioles and alveoli, accompanied by lymphocytes infiltrating the lamina propria of bronchioles. BALT hyperplasia leading to occlusion of bronchi and bronchioles, as well as perivascular lymphocytic infiltrates, were also common. Grading of Mh microscopic lesion progression included: grade I 9.8% (4/41), grade II 14.6% (6/41), grade III 31.7% (13/41), and grade IV 43.9% (18/41) (Figure 2). Four (9.8%) animals had only grade I lesions. Four (9.8%) animals had only grade II lesions. Two (4.9%) animals had a combination of grade I and grade II lesions. Grade III was observed singly in nine animals (22%); a combination of grade III, II and I lesions was noted in four (9.8%) animals. Fifteen (36.6%) animals had only grade IV lesions; a combination of grade IV and III lesions was seen in three (7.3%) animals.

Bronchointerstitial pneumonia was the only pneumonic pattern detected in 67.4% of animals (29/43) with CBP. In 27.9% (12/43) of examined lungs, these microscopic findings were accompanied by typical interstitial pneumonia. The main features observed in these cases were thickened alveolar septa due to infiltration of lymphocytes, plasma cells and macrophages, as well as type II pneumocyte hyperplasia. In addition, a mixture of desquamated epithelial cells and macrophages was usually present in the lumen of bronchiole and alveoli. Rarely, multinucleated giant cells were seen within the alveolar exudate.

Gross pneumonic patterns of fibrinonecrotic, embolic and interstitial pneumonic and/or pleuritis were confirmed histologically. The main histopathologic findings in FNP cases were fibrinocellular exudate composed of fibrin, neutrophils, macrophages and necrotic debris within the lower airways, as well as hemorrhage and vascular thrombosis. Marked fibrosis around areas of lung necrosis and thickening of visceral pleura by fibrous connective tissue were observed in chronic cases.

#### 3.1.3. Immunohistochemical Analysis

**Figure 3:**
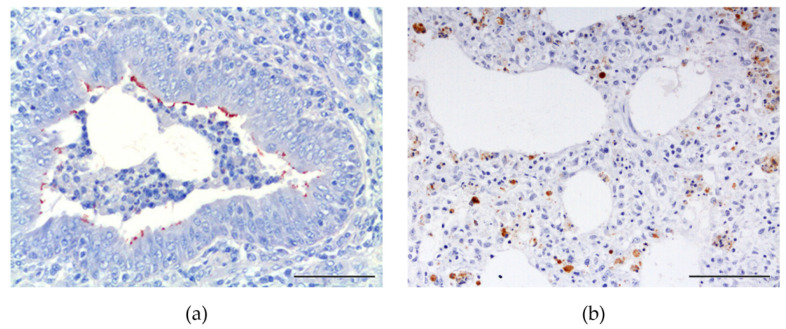
Immunolabeling for Mh and PCV2. (**a**) Granular positive immunolabeling to Mh-antigen on the luminal surface of the ciliated respiratory epithelium (IHC anti-Mh; ABC technique; AEC; magnification 200×). Scale, 100 µm. (**b**) Positive granular intracytoplasmic immunolabeling for PCV2 observed in alveolar macrophages (IHC anti-PCV2; ABC technique; AEC; magnification 200×). Scale, 100 µm.

Mh-antigen was detected along the respiratory epithelial surface of bronchi and bronchioles (Figure 3) in 70.7% (29/41) of the lungs with bronchointerstitial pneumonia. According to the microscopic lesion grade, immunolabeling was detected in different percentages: grade I (50%, 2/4); II (66.7%, 4/6); III (69.2%, 9/13) and IV (77.7%, 14/18). Furthermore, PCV2-antigen was detected in alveolar macrophages in 33.3% (4/12) of animals with microscopic evidence of bronchointerstitial and interstitial pneumonia patterns (Figure 3). In two cases (16.7%, 2/12), immunolabeling for PCV2 overlapped immunolabeling for Mh. IHC for PRRSV was negative.

### 3.2. Serologic Analysis

All animals were vaccinated against Mh at the age of 21 days. At 38 days of age (17 dpv), all animals lacked detectable maternal and post-vaccination anti-Mh antibodies; however, 45% (10/22) and 100% (22/22) of those were seropositive for PCV2 and PRRSV, respectively. All 77-day-old animals (56 dpv) lacked detectable anti-PCV2 antibodies; anti-Mh antibodies were detected in 18% (4/22) of the animals, and a slight decreased level in PRRSV antibodies (73%, 16/22) was registered. The 136-day-old group (115 dpv) showed 95% (21/22), 100% (22/22) and 68% (15/22) seropositivity for Mh, PRRSV and PCV2, respectively.

### 3.3. Evaluation of Productive Parameters

Average carcass weight (ACW) and age at slaughter in this cohort were 76.1 kg and 204.6 days, respectively. Animals without gross lesions were sent to slaughter with an ACW of 77.7 kg at 200.8 days old, whereas animals with lung disease had 75.5 kg ACW at 206 days old. While a significant difference was detected at the age at slaughter between the groups (*p* = 0.035), the ACW was not significantly dissimilar (*p* = 0.248).

Animals with CBP at 206.7 days old had an ACW of 75.5 kg and 16.4% of affected lung parenchyma. Animals with pleuritis at 206 days old had an ACW of 74.2 kg and 25.3% of affected lung parenchyma. Finally, animals with FNP at 201.4 days old had an ACW of 80.6 kg and 8.5% of affected lung parenchyma. However, no significant difference was detected between the groups (*p* = 0.571, *p* = 0.235, *p* = 0.823, respectively). Statistical analyses results are summarized in Table 2.

The DWG in animals without lesions was 567.2 gr/day, whereas in animals with CBP and pleuritis, it was 516.1 and 505.1 gr/day, respectively. Animals with CBP that had less than 10% of lung parenchyma affected were sent to slaughter at 204.6 days on average, weighing 77 kg, with a DWG of 531.3 gr/day. Animals with more than 10% of lung parenchyma affected were sent at 208.8 days old, with 74.1 kg and a DWG of 500.8 gr/day. Animals with pleuritis affecting less than 10% of the lung parenchyma had an ACW of 74.6 kg and a DWG of 509.9 gr/day, whereas animals with more than 10% weighed 73.7 kg and gained an average of 499.7 gr/day, with both groups slaughtered at an average of 206 days old.

When the age at slaughter was analyzed in animals with CBP, we observed that animals sent at a younger age (average of 189.2 days) had an average DWG of 577.3 gr/day, while animals sent later (215.8 day) had an average DWG of 473.7 gr/day. These groups presented an ACW and percentage of affected pulmonary parenchyma of 76.2 kg and 12.7%, and 75 kg and 18.3%, respectively. Microscopically, grade I and II bronchointerstitial pneumonia lesions were observed mainly in animals that were sent to slaughter at 189.2 days, while animals with CBP that remained in the farm for longer, i.e., 209.1 and 215.8 days of age, had grades III and IV, respectively.

## 4. Discussion

Respiratory diseases cause significant financial losses, representing one of the most important health and welfare issues in intensive pig farming worldwide. In this study, 73.1% of lungs showed some gross pneumonic pattern at slaughter. This percentage of inflammatory lung disease was higher than previous comparable studies, wherein lung lesions occurred in 49.7% and 67.1% of the animals studied [4,23]. Specifically, CBP was detected in nearly half of the inspected lungs (46.3%), which is similar to observations made by Merialdi et al. (2012) [10] and dissimilar from observations in other studies that reported lower or higher results (31–82%) [5,8,13,23,24,25]. The percentage of pulmonary parenchyma affected was 12.8% in lungs examined grossly and 16.4% in lungs with CBP lesions. The right lung lobes showed more lesions (9.6%) than the left (6.8%), which is likely due to the aerogenic Mh infection route and the presence of the cranial bronchus in the right lung [26,27,28]. CBP lesions are commonly associated with Mh infection; however, similar gross findings may be observed with other bacterial infections with or without concomitant viruses, for instance, *Pasteurella multocida* or SIV [29]. Mh-associated lesions may be accompanied or even masked by other inflammatory pneumonic patterns [11,24].

Pleuritis was common (17.6%) in the studied population. This result is within the wide range (6.3–26.8%) of pleuritis in slaughtered pigs [8,9,10,23] and contrasts with the high occurrence (45% or 65.1%) reported by some authors [24,25]. In pigs, pleuritis may be primary, often associated with *Haemophilus parasuis*, *Streptococcus suis* and *Mycoplasma hyorhinis* infection, or secondary, typically associated with *A. pleuropneumoniae* or Mh [24]. In our cases, Mh played a main role in development of lung lesions, and possibly pleuritis, based on immunohistochemical and serologic analyses results. However, the involvement of other pathogens cannot be ruled out. Fibrinonecrotic pneumonia compatible with *A. pleuropneumoniae* infection was observed in 6.5% of inspected lungs. This prevalence was lower than in other comparable studies, which reported 12.5% to 16.5% [4,23,24]. No bacteriologic analyses were performed in these cases; therefore, no further conclusions can be drawn about the etiology of these lesions.

Bronchointerstitial pneumonia compatible with Mh infection was detected in 95.3% of animals with CBP gross lesions. These lesions were characterized by mixed inflammatory infiltrates in bronchial, bronchiolar, and alveolar lumens, and were accompanied by lymphocytes infiltrating the lamina propria, and BALT hyperplasia in some cases. Bronchointerstitial pneumonia was the only pattern detected in 67.4% (29/43) of cases and was accompanied by typical interstitial pneumonia lesions in 27.9% (12/43). Pallarés et al. (2021) [23] described bronchointerstitial pneumonia in 78.2% of the animals analyzed and a combination of bronchointerstitial pneumonia and interstitial pneumonia in 6.7% of the cases.

Semiquantitative analysis of microscopic lesions associated with Mh has only been applied in experimental Mh infection [22,30,31]. In these fattening pigs with natural infection, chronic lesions (III and IV) were observed more often (31.7% and 43.9%) than early lesions (I and II) (9.8% and 14.6%), which is in agreement with the chronicity and lesion progression described in experimental studies [11,22,30,31]. Grade III and IV lesions were detected primarily in animals with CBP that remained on the farm for a longer period (209.1 and 215.8 days of age, respectively). Some fattening pigs exhibited early bronchointerstitial pneumonic lesions, singly (grade I or II lesions in 9.8%, for each one) or in combination with grade III (9.8%). These findings suggest that some animals suffered Mh infection or reinfection processes during the fattening phase, possibly due to Mh recirculation on the premises.

In this survey, the combination of bronchointerstitial and interstitial pneumonia was common (27.9%), and animals with both patterns had a higher average percentage of affected lung parenchyma (25%). Under field conditions, microscopic lung lesions often suggest Mh and viral pathogen coinfection in these cases [11,21]. Furthermore, previous studies described that the interaction between Mh and PRRSV or PCV2 exacerbated the severity of lung lesions when a sequential infection occurs. The latter could explain the high percentage of affected pulmonary parenchyma observed in these pigs [32,33,34].

Lung disease in swine is multifactorial and is often the result of the interplay of host, pathogen and environmental factors [7,8,9,10]. From a diagnostic standpoint, Mh culture is the gold standard; however, it is rarely used for routine diagnosis. Other available diagnostics may include immunofluorescence, IHC or in situ hybridization, although these tests have limited sensitivity and can only be made post mortem [3]. In the present study, positive anti-Mh immunolabeling was observed in 71% animals with bronchointerstitial pneumonia. Other authors described Mh-antigen detection in 61.9% of lung samples; less often, Mh-antigen is detected in naturally infected pigs [35]. The authors analyzed diseased lung samples that had been fixed in formalin for several years; this could explain the slightly lower results. Mh-antigen was more abundant in grade III and IV lesions (69.2% and 72.2%, respectively) than in earlier stages (50% of grade I and 66.7% of grade II). These results align with higher Mh detection by PCR in bronchi of naturally infected animals [11,36]. In experimental studies, other authors detected immunolabeling in 100% of infected animals by IHC, observing a weaker labeling at chronic stages [37].

PCV2-antigen was detected in 16.7% of the lung samples with bronchointerstitial and interstitial pneumonic lesions. This result is similar to a previous comparable study by Hansen et al. (2010) [11] in natural conditions, wherein the authors described positively labeled lungs in 14% of cases with porcine respiratory disease complex, analyzed at slaughter. PCV2 has a marked lymphoid tropism; often, PCV2-antigen is present in smaller amounts and lower intensity in lung sections. In addition, PCV2 detection by IHC is less sensitive than PCR analysis [38,39]. These two animals also had detectable Mh-antigen, which suggests dual infection. IHC for PRRSV was negative in all animals tested; however, several animals were seropositive. Stage of infection, age, lung anatomic location tested (twice as often in anterior and middle lung lobes as in caudal lobes), number of lung tissue sections analyzed, and suboptimal or prolonged fixation may have influenced pulmonary virus load and laboratorial detection of PRRSV by IHC [40,41,42,43].

Serologic assays are commonly used to monitor the health status of swine herds. ELISA testing may provide useful information on the presence of passive (e.g., maternal) and active (e.g., infected and vaccinated) immunity and allow for seroconversion monitoring [6]. All animals in this study were vaccinated against Mh at 21 days of age; they lacked maternal and post-vaccination anti-Mh antibodies at 38 days of age (17 dpv). Anti-Mh antibodies were detected in 18% and 95% of the animals at 77 (56 dpv) and 136 days of age (115 dpv), respectively. The lack of maternal anti-Mh antibodies observed at 38 days old was in agreement with Flores et al. (2006) (0–3.4% of piglets at 42 days old) [44]. In our study, Mh antibodies were detected at a later period (56 dpv) and in a low percentage (18%), indicating that many animals were unprotected at the time of greatest susceptibility. Other studies described post-vaccination seroconversion in 29.4% at 21 dpv with a single dose of commercial inactivated Mh vaccine at 21 days old [45] or in 44.8% at 42 dpv and 68.9% at 70 dpv with a single dose bacterin at 42 days old [44]. This delayed seroconversion could be due to several factors, such as age at immunization, inadequate vaccine management or viral coinfections such as PRRSV [46,47,48,49], with the latter as the most likely factor in our study. In addition, the ELISA test used does not discriminate between vaccination and infection-associated antibodies.

Anti-PRRSV antibodies were detected in 100% of the animals at 38 days of age; seropositivity decreased mildly (73%) and returned to 100% at 77 and 136 days of age, respectively. The maternal anti-PRRSV antibodies declined over time after weaning (30 days old) to a serologic PRRSV-negative status at 70 days old [50]. The detection of high anti-PRRSV titers at different time points suggested PRRSV continuous circulation in the farm [7,51], although there was no IHC etiologic confirmation.

Anti-PCV2 antibodies were detected at 38 days of age (45%) and were not detectable in any of the animals at 77 days old. At 136 days of age, 65% of the animals were seropositive for PCV2 again. Maternal antibodies may prevent development of post-weaning multisystemic wasting syndrome, as observed in experimental infections [52]. A remarkable reduction of anti-PCV2 maternal antibodies between 38 and 77 days old was detected, which is in agreement with previous studies in which a gradual decrease in antibody titers from 21 up to 49–77 days of age was shown [53,54]. The seroconversion observed at 136 days old (68%) can be interpreted as infection. Similar results have been described in seroconversion against PCV2 at 105–112 to 196 days old by other authors [25,53,55,56].

Environmental factors and management practices play important roles in swine respiratory disease occurrence [7,8,9,10,11]. In this study, the natural ventilation and lack of temperature control in fattening pens could have contributed to infection and reinfection processes and could explain the incidence of lesions observed at the slaughterhouse. Regardless of intrinsic and extrinsic factors resulting and modulating pulmonary disease, production parameters and carcass quality are significantly negatively affected by greater volume of lung parenchyma affected [3]. The associations between lung lesions with specific pneumotropic pathogens and impacts on productive parameters in swine have been extensively studied [3,4,5,8,9,10,11,13,23,24,57,58]. However, most of these studies have not deepened on lesion chronology with emphasis on Mh-associated lesion development and scoring. Furthermore, few studies have analyzed these lesions while knowing the exact age of the animals. By contrast, we applied a semiquantitative analysis system developed under experimental settings [22,30,31], which enabled us to score and infer lesion chronology and disease progression in natural infection. This approach allowed us to identify reinfection events, including near-to-slaughter reinfections and to confirm endemic circulation in the farm.

In our study, animals without lung lesions were sent at slaughter with an ACW of 77.7 kg at 200.8 days. The carcasses of animals with CBP (75.5 kg) weighed less than animals without lesions and those slaughtered at older ages (206 day-old); this was also noted in animals with pleuritis (74.2 kg at 206 day-old). No significant difference was detected in the weight of carcasses between the animals with and without lesions, which is expected because that this was one of the farmer’s primary criteria to slaughter the animals. However, these values may be economically relevant. There was almost 3.5 kg of difference between the groups, suggesting substantial economic losses in the sale of the carcasses. In addition, these animals remained almost 6 more days on the farm, which increased economic losses. Animals with CBP showed lesions affecting an average of 16.7% of parenchyma. Those that had less than 10% were slaughtered at an average age of 204.6 days and an ACW of 77 kg. Comparatively, animals with more than 10% of lung parenchyma affected were sent at a later time (208.8 day-old) with a lower ACW (74.1 kg). In addition, these animals had a lower DWG than animals with less than 10% and animals without lesions (500.8, 531.3 and 546.9 gr/day, respectively). This is in agreement with other studies that found a reduction of 37.5–41 gr/day or a 9.3% reduction in their growth when Mh-associated lesions exceeded 10% [57,59]. Other studies have suggested a decrease in animal weight associated with the extent of lung consolidation, with 0.7 to 1.8 gr for each 1% of lung parenchyma affected [5,13,58] and a reduction on growth rate of up to 7% at slaughter [58]. The presence of severe lung lesions in pigs at slaughter frequently results in reduced liveweight [4,60,61]. Furthermore, we conducted a virtual simulation (https://www.3tres3.com/simulador-de-costes/; accessed on 17 November 2021) to estimate and compare economical gains and losses by modifying the variable “age at slaughter”, based on the results of this study. These calculations indicated a potential loss of up to EUR 9 per pig at slaughter. This result is comparable to other studies, for instance, Ferraz et al. (2020) [5], in which the authors estimated a loss of USD 6.55 per pig at slaughter in the group of pigs with more than 15.1% of lesions. Moreover, high pneumonia prevalence and high lung lesion scores may result in negative effects on pork quality by leading to changes in pH values (e.g., pale, soft, exudative (PSE) meat), water-holding capacities, color, flavor, and cooking quality loss [60,62]. These negative impacts were not addressed in our study.

Vaccination against Mh is common practice in pig herds worldwide. This reduces clinical signs and severity of lung lesions and seeks to improve DWG and feed conversion ratio, shortening the time to reach slaughter weight [5,25,49,63,64]. The partial protection observed in our study, influenced by the participation of other respiratory pathogens and management factors, could explain the decreased growth performance.

Furthermore, animals with pleuritis also had lower carcass weight and were older at slaughter (74.2 kg at 206 days old) than animals without lesions (77.7 kg at 200.8 days old). Permentier et al. (2015) [60] and Karabasil et al. (2017) [4] described a reduction in the hot carcass weight in animals with moderate to severe pleuritic lesions. In our study, animals with more than 10% weighed 73.7 kg, with a DWG of 499.7 gr/day. The observed pleuritides were compatible with chronic lesions by *A. pleuropneumoniae*; however, no bacteriologic confirmation was pursued in these cases. The presence of *A. pleuropneumoniae* is often associated with severe economic losses, high morbidity and mortality, decreased rate of weight gain, inefficient feed conversion, and increased time to market in chronically infected herds [65].

## 5. Conclusions

Respiratory disease is a multifactorial disease process of major relevance to the swine industry worldwide. The multiple etiologies vary by country, region and production system, and their exact pathogenic roles are often unknown. In the present study, a naturally infected herd was evaluated using histopathologic, immunohistochemical and serologic diagnostic tools. CBP lesions compatible with Mh infection were most common; histopathologic findings suggested Mh reinfection events. IHC analysis confirmed coinfections by Mh and PCV2. There was serologic evidence of Mh, PCV2, and PRRSV exposure. Furthermore, the animals studied showed low post-vaccination immune response against Mh, probably due to concomitant PRRSV infection at vaccination time. This study confirms the negative impact of lung disease on ACW and DWG, with prolonged finishing periods. In light of these results, we suggest that animals that do not reach the weight at slaughter should be sent to slaughter in order to avoid further negative impacts of respiratory diseases, including reinfections, on productive parameters. The combination of pathologic, immunohistochemical, and serologic examinations may be of high value to characterize the etiologic signature of respiratory processes in swine, therefore aiding in management practices and medical decisions.

## Figures and Tables

**Table 1:** Summary of gross examinations at the slaughterhouse.

Variable	Category	Frequency (%)	*p* Value
Gross lesions (*n* = 108)	Animals with lesions	79 (73.1%)	< 0.001
	Animals without lesions	29 (26.9%)	
Lung lesion patterns ^1^ (*n* = 79)	CBP	50 (63.3%)	< 0.001
	Pleuritis	19 (24.1%)	
	FNP	7 (8.9%)	
	IP	2 (2.5%)	
	EP	1 (1.3%)	
Percentage of affected lung parenchyma ^1^ (*n* = 79)	(0.1–10%)	41 (51.9%)	< 0.001
	(10.1–25%)	23 (29.1%)	
	(25.1–50%)	7 (8.9%)	
	(50.1–75%)	2 (2.5%)	
	(75.1–100%)	6 (7.6%)	

^1^ The variable has been summarized for animals with lesions (*n* = 79). CBP, cranioventral bronchopneumonia; FNP, fibrinonecrotic pneumonia; IP, interstitial pneumonia; EP, embolic pneumonia.

**Table 2:** Comparison of the general characteristics of the different groups, according to the presence or absence of macroscopic lesions, as well as the different most frequent patterns.

		Global Sample (*n* = 108)			Animals with Lesions ^1^ (*n* = 76)			
Variable	Statistic	Animals without Lesions (*n* = 29)	Animals with Lesions (*n* = 79)	*p* Value	CBP (*n* = 50)	Pleuritis (*n* = 19)	FNP (*n* = 7)	*p* Value
Age of slaughter (days)	Mean (SD)	200.8 (12.4)	206 (11.4)	0.035	206.7 (11)	206 (12.2)	201.4 (15.1)	0.571
Carcass weight (kg)	Mean (SD)	77.7 (9.5)	75.5 (8.5)	0.248	75.5 (9)	74.2 (7.7)	80.6 (6.7)	0.235
Affected lung parenchyma(%)	Mean (SD)	-	17.5 (25.3)	-	16.4 (22.7)	25.3 (35.1)	8.5 (7.2)	0.823

^1^ Statistical analyses for those categories that were represented by at least seven animals. SD, standard deviation; CBP, craneoventral bronchopneumonia; FNP, fibrinonecrotic pneumonia.

## Data Availability

The data presented in this study are available on request from the corresponding author. The data are not publicly available due to privacy.
